# Heat Stress Affects Jejunal Immunity of Yellow-Feathered Broilers and Is Potentially Mediated by the Microbiome

**DOI:** 10.3389/fphys.2022.913696

**Published:** 2022-05-23

**Authors:** Wen-Chao Liu, Meng-Yi Huang, Balamuralikrishnan Balasubramanian, Rajesh Jha

**Affiliations:** ^1^ Department of Animal Science, College of Coastal Agricultural Sciences, Guangdong Ocean University, Zhanjiang, China; ^2^ Department of Food Science and Biotechnology, College of Life Science, Sejong University, Seoul, South Korea; ^3^ Department of Human Nutrition, Food and Animal Sciences, College of Tropical Agriculture and Human Resources, University of Hawaii at Manoa, Honolulu, HI, United States

**Keywords:** gene expression, gut microbiota, heat stress, intestinal immunity, yellow-feathered broilers

## Abstract

In the perspective of the global climate change leading to increasing temperature, heat stress (HS) has become a severe issue in broiler production, including the indigenous yellow-feathered broilers. The present study aimed to investigate the effects of HS on jejunal immune response, microbiota structure and their correlation in yellow-feathered broilers. A total of forty female broilers (56-days-old) were randomly and equally divided into normal treatment group (NT group, 21.3 ± 1.2°C, 24 h/day) and HS group (32.5 ± 1.4°C, 8 h/day) with five replicates of each for 4 weeks feeding trial. The results showed that HS exposure increased the contents of TNF-α, IL-1β, and IL-6 in jejunal mucosa (*p* < 0.05). The HS exposure up-regulated the relative fold changes of *NF-κB, TNF-α, IL-1β,* and *IL-6* (*p* < 0.01) while down-regulated the relative fold change of *IFN-γ* in jejunal mucosa (*p* < 0.05)*.* Meanwhile, HS had no significant impacts on alpha diversity of jejunal microbiota such as Simpson, Chao1 richness estimator (Chao 1), abundance-based coverage estimators (ACE), and Shannon index (*p* > 0.10). Broilers exposed to HS reduced the jejunal microbial species number at the class and order level (*p* < 0.05). Moreover, HS decreased the relative abundance of *Ruminococcus*, *Bdellovibrio,* and *Serratia* at the genus level in jejunum (*p* < 0.05). At the phylum level, four species of bacteria (*Bacteroidetes, Cyanobacteria, Thermi,* and *TM7*) were significantly associated with immune-related genes expression (*p* < 0.05). At the genus level, ten species of bacteria were significantly correlated with the expression of immune-related genes (*p* < 0.05), including *Caulobacteraceae, Actinomyces, Ruminococcaceae, Thermus, Bdellovibrio, Clostridiales, Sediminibacterium, Bacteroides, Sphingomonadales* and *Ruminococcus*. In particular, the microbial with significantly different abundances, *Ruminococcus* and *Bdellovibrio*, were negatively associated with pro-inflammatory cytokines expression (*p* < 0.05). These findings demonstrated that HS exposure promoted the production of pro-inflammatory cytokines in yellow-feathered broilers’ jejunum. The detrimental effects of HS on jejunal immune response might be related to dysbiosis, especially the reduced levels of *Ruminococcus* and *Bdellovibrio*.

## Introduction

There is an increasing pursuit of flavor in the huge chicken consumer market in China and almost half of Chinese chicken meat is from locally yellow-feathered broilers because of their good meat quality (Gou et al., 2016). South China is the major production area for yellow-feathered broilers (Wang Y. et al., 2021). However, South China is in tropical and subtropical regions, and heat stress (HS) has become a great challenge for yellow-feathered broiler production (Liu et al., 2019). The HS exposure results in detrimental impacts on growth performance and has been well characterized, such as reduced feed intake, poor feed efficiency and decreased growth rate (Chauhan et al., 2021). Along with reduced growth rate, HS induces multiple deleterious consequences on physiological homeostasis, systemic immune function, metabolism status, and gut health (Guo et al., 2021a; Guo et al., 2021b; Liu et al., 2021a; Wasti et al., 2021a). Hence, HS has been one of the most harmful environmental stressors in broiler production, including the indigenous broilers, which causes large economic losses annually (Lara and Rostagno, 2013; Liu et al., 2019).

The gastrointestinal tract (GIT) is particularly vulnerable to HS, because the vasoconstriction in broiler’s GIT is increased to redistribute blood to the peripheral circulation, to maximize heat dissipation under HS situation (Vandana et al., 2021). Thus, HS leads to ischemia-hypoxic injury of the intestinal epithelial barrier and compromises gut integrity (Quinteiro-Filho et al., 2010). Intestinal mucosal immunity has attracted extensive attention for its effectiveness in strengthening the gut barrier function and protecting against pathogenic infection (Kogut and Arsenault, 2016). It has been found that HS-induced gut damage is partly attributed to the inflammatory response, such as an increase of pro-inflammatory cytokines production in broiler’s intestines (Song et al., 2017; Wu et al., 2018). On the other hand, the gut microbiota has a variety of biological functions, which is not only involved in the digestion of nutrients but also plays a vital role in maintaining intestinal barrier integrity and modulating the gut immune function of broilers (Yadav and Jha, 2019). With the development of high-throughput sequencing technology, emerging evidence demonstrated that HS disrupted the community structure and composition of hindgut (ileal and cecal) microbiota in broilers (Wang et al., 2018; Shi et al., 2019; Wang G. et al., 2021); but limited information is available about the effects of HS on fore-midgut microbiota in broilers. As the middle segment of the small intestine, the immune and microbial barrier of jejunum is critical for gut health in broilers (Rajput et al., 2017; Li et al., 2019). Furthermore, compared to the fast-growing chickens, including Ross and Cobb broilers, although the yellow-feathered broilers have better heat tolerance due to their slower growth and lower metabolic rate; it has been confirmed that HS also has negative impacts on gut health in this type of broilers (Gou et al., 2016; Liu et al., 2021a). However, there is a lack of information on changes in jejunal immunity, and the microbiome of yellow-feather broilers under HS. In this context, the present study was done to evaluate the effects of HS on jejunal immune response, microbial community, and their correlation in yellow-feathered broilers. Thus, it can provide novel insight into the interaction between the jejunal microbiome and immune response in slow-growing broilers under HS.

## Materials and Methods

### Animals, Experimental Design, and Management

A total of forty female Huaixiang chickens (eight-week-old, slow-growing type Chinese yellow-feathered broilers) were used in a 4 weeks HS trial (9–12 weeks of age during the study). The birds with an initial average body weight (BW) of 840.75 ± 20.79 g were sourced from a local supplier (Zhanjiang, Guangdong, China). The age selection of broilers was made considering the indigenous broilers entering the growing-finishing stage after 8-weeks of age, and growing-finishing broilers are more prone to HS for production (Wasti et al., 2020). The female broilers were only used to exclude the influence of gender on the study. It was also because the local people prefer to consume female yellow-feather broilers (Lin, 2008). Chickens were randomly and equally divided into two treatments in a completely randomized design. The treatments included the normal treatment group (NT) (21.3 ± 1.2°C throughout the experimental period, thermoneutral zone) and HS group (32.5 ± 1.4°C, 8 h/day, from 9:00 am to 17:00 pm). The ambient temperature for the rest of the time of the HS group was the same as the NT group. The relative humidity of NT and HS were maintained at 55–70%. Each group had five replications with four broilers per replicate, and one cage was used as one replicate. The chickens were kept in three-layer wire cages (one cage on top, middle, and bottom, with 20 cm gap between each layer for collecting and cleaning excreta). The size of each cage was 90 (length) × 70 (width) × 40 (height) cm. Plastic trays were used under each cage to collect the excreta, and the excreta were manually cleaned twice a day. The birds were ensured that all chickens had free access to water and feed. All broilers were fed corn-soybean meal basal diet to meet the nutrient requirements recommended by the Chinese chicken breeding standard (NY/T33-2004). The environmental control equipment, diet’s composition, and nutrient levels were as reported in our previous study (Guo et al., 2021a).

### Sample Collection

At the end of the HS trial, one chicken was randomly selected from each replicate and sacrificed by neck bloodletting (*n* = 5/group). The jejunum was rapidly separated, and then approximately 2 g of jejunal digesta samples were collected and preserved in the liquid nitrogen and subsequently used for 16S rRNA sequencing. Afterward, the jejunum was opened, and the digesta was washed with PBS at 4°C; the jejunal mucosa was scraped off by glass slides and frozen in liquid nitrogen and then stored at −80°C for further detection of cytokines content and immune-related gene expression.

### Determination of Jejunal Cytokines Concentration

The tumor necrosis factor-α (TNF-α), interferon-γ (IFN-γ), interleukin (IL)-1β, IL-2, IL-4, IL-6, and IL-10 concentrations in jejunal mucosa samples, were analyzed using commercial cytokines ELISA kits of chickens (Catalog numbers: TNF-α, MM-0938O2; IFN-γ, MM-0520O1; IL-1β, MM-36910O2; IL-2, MM-0528O2; IL-4, MM-0527O2; IL-6, MM-0521O2; IL-10 MM-1145O2; Jiangsu Enzyme Immunology Co., Ltd., Suzhou, China) following the manufacturer’s instructions.

### Detection of Jejunal Immune-Related Genes Expression

Total RNA was extracted from the jejunal mucosa samples using RNA extraction kits (Catalog No. N066, Jiancheng Bioengineering Institute, Nanjing, China). The reverse transcription of cDNA from RNA using RT reagent kits (Catalog No. RR047A, TaKaRa Biotechnology Co., Ltd, Dalian, China). Then the quantitative real-time PCR (qPCR) was performed to detect the mRNA expression of immune-related genes, including *NF-κB, TNF-α, IFN-γ, IL-1β, IL-2, IL-4, IL-6, and IL-10*. The CFX-96 real-time PCR detection system (BioRad, Irvine, CA, United States) was used for the qPCR reaction. The specific primers, reaction system, and conditions were similar to our previous study (Liu et al., 2021b). β-actin was used as an internal reference gene. The mRNA expression levels of the jejunal immune-related genes were calculated using the 2^−ΔΔCt^ method (Livak and Schmittgen, 2001), and the data were expressed as a relative fold change to the average value of the NT group.

### Jejunal Microbiome Analysis

The total microbial DNA of jejunal digesta was extracted using QIAamp DNA Stool Mini Kit (Code No. 51306, QIAGEN, CA, Hamburg, Germany). This study used the Illumina Miseq Platform of Personalbio Technology Co., Ltd. (Shanghai, China) to perform paired-end sequencing of V3-V4 region of 16S rRNA gene. The primer pairs were 338F (5’-ACT​CCT​ACG​GGA​GGC​ACA​G-3’) and 806R (5’- GGACTACHVGGGTWTCTAAT-3’). The processing and analysis of sequencing data was performed using QIIME2 (Quantitative Insights Into Microbial Ecology 2) 2019.4 and according to the official tutorials (https://docs.qiime2.org/2019.4/tutorials/). Briefly, using cutadapt (v2.3) to excise the primer fragments of the raw sequencing reads and to remove the unmatched sequences with the primers. The fastq_mergepairs module of Vsearch (v2.13.4) was used to assemble the sequences; using the fastq_filter module of Vsearch (v2.13.4) to perform quality control on the assembled sequences; the derep_fulllength module of Vsearch (v2.13.4) was used to remove the duplicates sequences, and the uchime_denovo module of Vsearch (v2.13.4) was conducted to remove the chimeras, thereby resulting in high-quality sequences. Subsequently, the high-quality sequences are clustered at the 97% similarity level using the cluster_size module of Vsearch (v2.13.4), and the operational taxonomic units (OTUs) were obtained accordingly. The bacterial taxonomic information corresponding to each OTU was obtained based on the Greengenes database (Release 13.8, http://greengenes.secondgenome.com/).

### Statistical Analysis

The data on jejunal cytokines levels, immune-related genes expression, and the difference in the jejunal microbiota were analyzed using SAS 9.4 (SAS Institute Inc., Cary, NC). Student’s t-test was performed to compare the differences between the two groups. Results are expressed as mean ± standard error. The alpha diversity, beta diversity, and the composition of jejunal microbiota were analyzed using Gene Cloud Analysis Platform (based on the kernel of QIIME 2, https://www.genescloud.cn) of Personalbio Technology Co., Ltd. (Shanghai, China). The Spearman correlation analysis was performed to determine the association between the jejunal microbiota (at the phylum and genus level) and immune-related genes expression using online software (based on the kernel of QIIME 2, https://www.genescloud.cn) of Personalbio Technology Co., Ltd. (Shanghai, China). The probability (*P*) < 0.05 was considered to be significant, and 0.05 ≤ *p* < 0.10 was considered to be a trend of significance.

## Results

### Jejunal Cytokines Content

The jejunal cytokines concentration treatment groups are presented in Table 1. Compared to NT group, broilers in HS group had higher levels of TNF-α, IL-1β, and IL-6 in jejunal mucosa (*p* < 0.05). There were no significant differences between NT and HS groups in jejunal INF-γ, IL-2, IL-4, and IL-10 contents (*p* > 0.10).

**TABLE 1:** Effects of heat stress on cytokines levels in jejunal mucosa of yellow-feathered broilers (*n* = 5).

Items	NT group	HS group	*p*-value
TNF-α, pg/mg protein	136.52 ± 8.76	188.87 ± 10.09	0.005
INF-γ, pg/mg protein	388.10 ± 8.59	376.65 ± 15.51	0.533
IL-1β, pg/mg protein	430.44 ± 7.36	481.72 ± 14.92	0.015
IL-2, ng/mg protein	4.76 ± 0.19	4.37 ± 0.38	0.392
IL-4, ng/mg protein	79.60 ± 4.98	87.14 ± 5.32	0.331
IL-6, pg/mg protein	59.76 ± 6.41	79.47 ± 4.77	0.039
IL-10, ng/mg protein	420.21 ± 8.89	437.65 ± 4.62	0.121

NT, normal treatment group, the broilers reared at 21.3 ± 1.2°C throughout the experimental period; HS, the broilers reared at 32.5 ± 1.4 C for 8 h/day (9:00 am to 17:00 pm); the abbreviations for the detected parameters (TNF-α, IFN-γ and IL) are tumor necrosis factor-α, interferon-γ and interleukin, respectively.

### Jejunal Immune-Related Genes Expression

**FIGURE 1:**
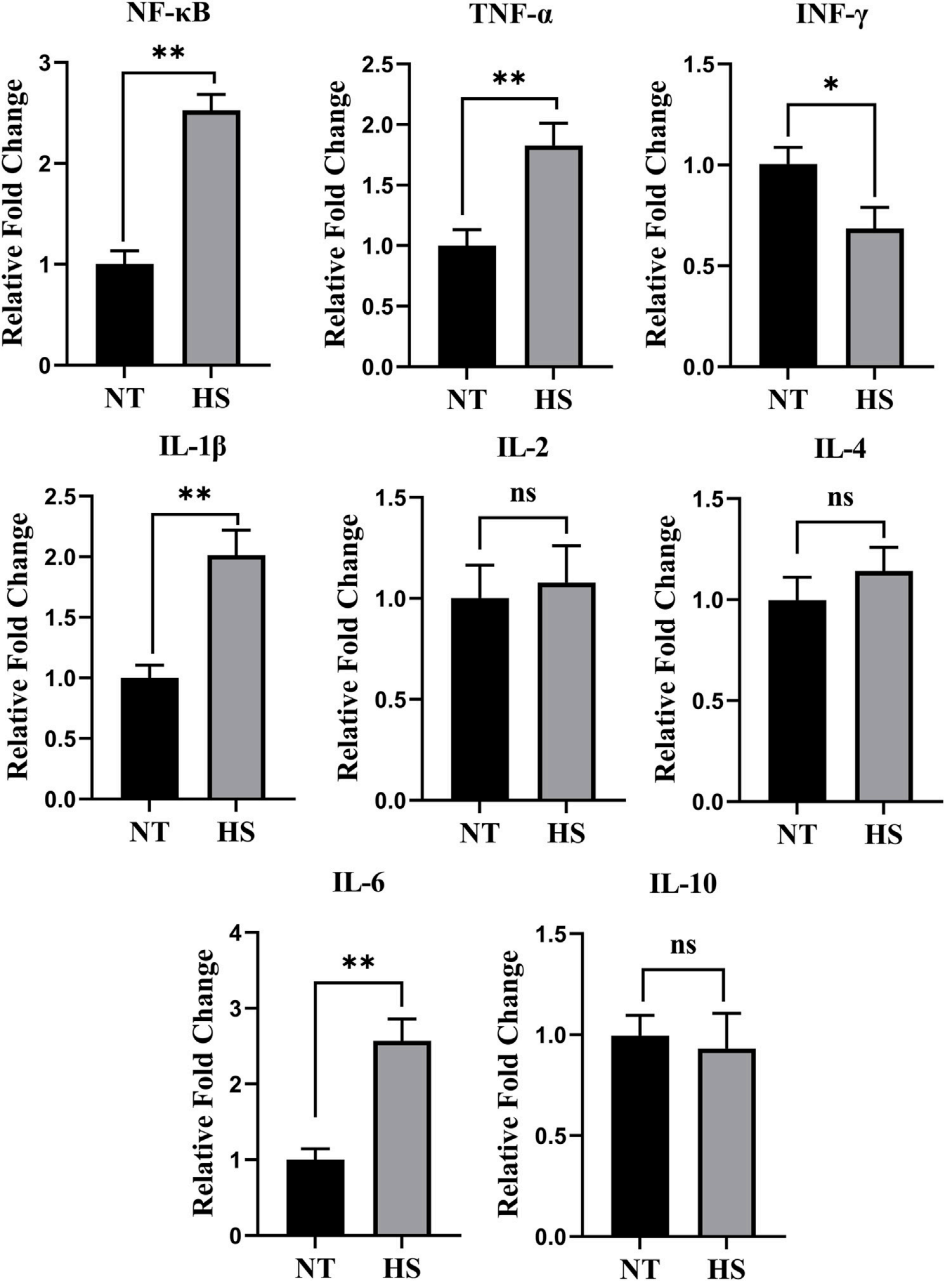
Effects of heat stress on relative fold change of jejunal immune-related genes in yellow-feathered broilers (*n* = 5). NT, normal treatment group (21.3 ± 1.2°C, 24 h/day); HS, heat stress group (32.5 ± 1.4°C, 8 h/day); the abbreviations for the detected parameters (NF-κB, TNF-α, IFN-γ and IL) are nuclear factor-kappa B, tumor necrosis factor-α, interferon-γ and interleukin, respectively. *p < 0.05, **p < 0.01, ^ns^no significant difference.

As illustrated in Figure 1, broilers exposed to HS up-regulated the relative fold changes of *NF-κB, TNF-α, IL-1β,* and *IL-6* (*p* < 0.01) while down-regulated the relative fold changes of *IFN-γ* in jejunal mucosa (*p* < 0.05)*.* In addition, the HS exposure had no significant impact on jejunal relative fold changes of *IL-2, IL-4, and IL-10* (*p* > 0.10).

### Microbial Diversity and Composition in Jejunum

**FIGURE 2:**
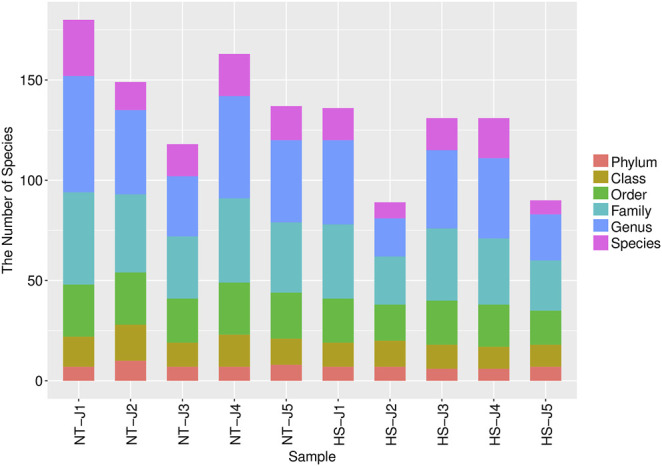
Effects of heat stress on distribution of jejunal microbial species number at each classification level in yellow-feathered broilers (*n* = 5). NT, normal treatment group (21.3 ± 1.2°C, 24 h/day); HS, heat stress group (32.5 ± 1.4°C, 8 h/day).

As shown in Table 2, the HS exposure had no significant effects on the alpha diversity of jejunal microbiota, including Simpson, Chao1 richness estimator (Chao 1), abundance-based coverage estimators (ACE), and Shannon index (*p* > 0.10). However, as presented in Table 3 and Figure 2, the HS exposure reduced the jejunal microbial species number at the class and order level (*p* < 0.05) and had a significant trend to decrease the jejunal microbial species number at the phylum and family level (*p* < 0.10).

**TABLE 2:** Effects of heat stress on alpha diversity of jejunal microbiota in yellow-feathered broilers (*n* = 5).

Items	NT group	HS group	*p-*value
Simpson	0.85 ± 0.43	0.88 ± 0.03	0.568
Chao 1	523.07 ± 38.30	552.37 ± 59.17	0.688
ACE	544.31 ± 41.30	573.16 ± 60.74	0.705
Shannon	4.71 ± 0.41	5.00 ± 0.32	0.594

NT, normal treatment group, the broilers reared at 21.3 ± 1.2 C throughout the experimental period; HS, the broilers reared at 32.5 ± 1.4 C for 8 h/day (9:00 am to 17:00 pm); Chao1, Chao1 richness estimator; ACE, abundance-based coverage estimators.

**TABLE 3:** Effects of heat stress on jejunal microbial species number at each classification level in yellow-feathered broilers (*n* = 5).

Items	NT group	HS group	*p-*value
Phylum	7.80 ± 0.58	6.6 ± 0.25	0.094
Class	14.80 ± 1.07	11.80 ± 0.37	0.029
Order	24.60 ± 0.87	20.00 ± 1.05	0.010
Family	38.60 ± 2.62	31.00 ± 2.74	0.080
Genus	44.40 ± 4.76	32.60 ± 4.80	0.119
Species	19.20 ± 2.48	13.40 ± 2.52	0.140

NT, normal treatment group, the broilers reared at 21.3 ± 1.2 C throughout the experimental period; HS, the broilers reared at 32.5 ± 1.4 C for 8 h/day (9:00 am to 17:00 pm).

**FIGURE 3:**
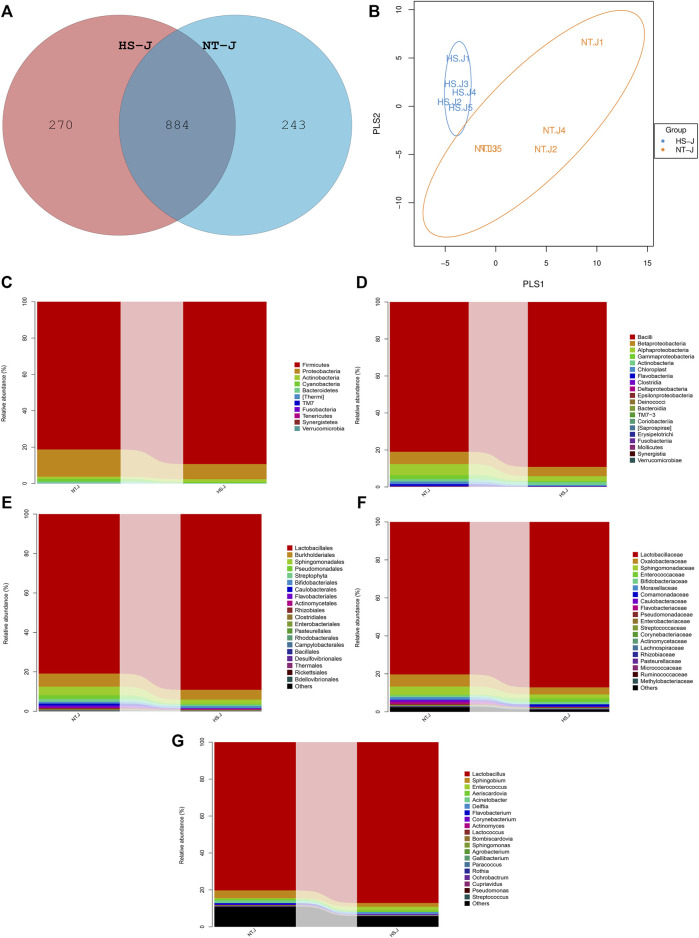
Effects of heat stress on jejunal microbial community in yellow-feathered broilers (*n* = 5). NT, normal treatment group (21.3 ± 1.2°C, 24 h/day); HS, heat stress group (32.5 ± 1.4°C, 8 h/day); **(A)**, operational taxonomic units (OTUs) venn diagram of jejunal microbiota between NT and HS groups; **(B)**, beta diversity of jejunal microbiota between NT and HS groups; **(C)**, microbial compositions at the phylum level; **(D)**, microbial compositions at the class level; **(E)**, microbial compositions at the order level; **(F)**, microbial compositions at the family level; **(G)**, microbial compositions at the genus level.

**FIGURE 4:**
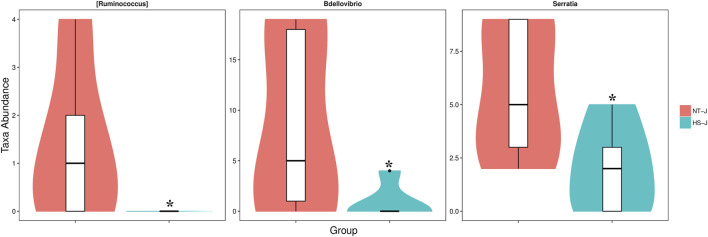
Microbials with significantly differential abundance at the genus level as affected by heat stress in yellow-feathered broilers (*n* = 5). NT, normal treatment group (21.3 ± 1.2°C, 24 h/day); HS, heat stress group (32.5 ± 1.4°C, 8 h/day) *p < 0.05.

The results of the jejunal microbial community are presented in Figures 3, 4. There were 884 commonly owned OTUs between the NT and HS groups, 243 OTUs of jejunal microbiota were unique in the NT group, and 270 OTUs of jejunal microbiota were unique in the HS group. The beta diversity revealed that the samples in NT and HS groups have different clusters. Besides, there were no significant differences in the relative abundance of each microbial at the phylum, class, order, and family level (*p* > 0.10). However, the HS exposure significantly reduced the jejunal relative abundance of *Ruminococcus*, *Bdellovibrio,* and *Serratia* at the genus level (*p* < 0.05).

### Correlation Between Jejunal Microbiome and Immune-Related Genes Expression

**FIGURE 5:**
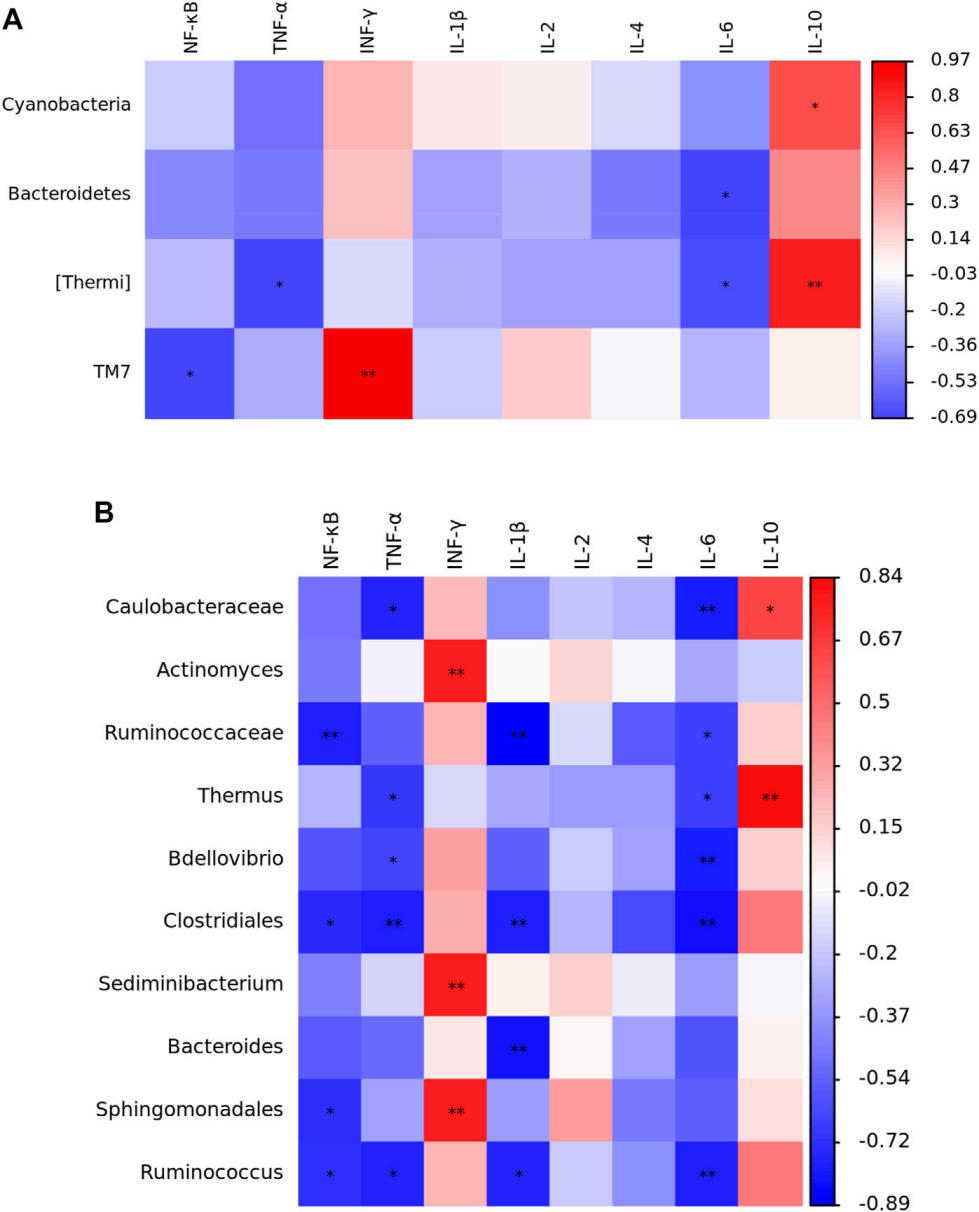
Spearman correlation analysis between the jejunal microbials **(A)**, phylum; **(B)**, genus level and immune-related genes expression in yellow-feathered broilers under heat stress (*n* = 5). NT, normal treatment group (21.3 ± 1.2°C, 24 h/day), HS, heat stress group (32.5 ± 1.4°C, 8 h/day); NF-κB, nuclear factor-kappa B; TNF-α, tumor necrosis factor-α; IFN-γ, interferon-γ; IL, interleukin. *p < 0.05, **p < 0.01.

The results of Spearman correlation analysis are showed in Figure 5. At the phylum level, *Cyanobacteria* was positively correlated with *IL-10* expression (*p* < 0.05). *Bacteroidetes* was negatively correlated with *IL-6* expression (*p* < 0.05). *Thermi* was negatively correlated with *TNF-α* and *IL-6* expression (*p* < 0.05), while positively correlated with *IL-10* expression (*p* < 0.01). *TM7* was negatively correlated with *NF-κB* expression (*p* < 0.05), but positively correlated with *IFN-γ* expression (*p* < 0.01). At the genus level, *Caulobacteraceae* was negatively correlated with *TNF-α* and *IL-6* expression (*p* < 0.05), whereas positively associated with *IL-10* expression (*p* < 0.05). *Actinomyces* and *Sediminibacterium* were positively related with *IFN-γ* expression (*p* < 0.01). *Ruminococcaceae* was negatively correlated with *NF-κB, IL-1β* and *IL-6* expression (*p* < 0.05). *Thermus* was negatively correlated with *TNF-α* and *IL-6* expression (*p* < 0.05), but positively correlated with *IL-10* expression (*p* < 0.01). *Bdellovibrio* was negatively correlated with *TNF-α* (*p* < 0.05) and *IL-6* (*p* < 0.01) expression. *Clostridiales* was negatively related with *NF-κB* (*p* < 0.05)*, TNF-α* (*p* < 0.01)*, IL-1β* (*p* < 0.01) and *IL-6* (*p* < 0.01) expression. *Bacteroides* was negatively associated with *IL-1β* expression (*p* < 0.01). *Sphingomonadales* was negatively correlated with *NF-κB* expression (*p* < 0.05), but positively related with *IFN-γ* expression (*p* < 0.01). *Ruminococcus* was negatively related with *NF-κB* (*p* < 0.05)*, TNF-α* (*p* < 0.05)*, IL-1β* (*p* < 0.05) and *IL-6* (*p* < 0.01) expression.

## Discussion

Due to the global climate change, high ambient temperature (around 32°C) often occurs in chicken houses during summer, thus impairing the health and productivity of broilers and resulting in significant economic losses to producers (Wasti et al., 2020; Greene et al., 2021). Although the yellow-feathered broilers have better thermal tolerance than fast-growing broilers due to their slower growth and lower metabolic rate, 32°C is not in the thermoneutral zone of slow-growing broilers, which also causes a series of deleterious consequences for yellow-feathered broilers (Shao et al., 2018; Guo et al., 2021a; Liu et al., 2021b). The gut is considered the main target of HS (Chauhan et al., 2021). The jejunum is the middle intestine and is related to most of the nutrients’ digestion and absorption in broilers; accordingly, the jejunum is more susceptible to HS (Song et al., 2013). Furthermore, as a representative segment of the small intestine, the jejunum is always selected for intestinal research, and jejunal immunity plays a crucial role in maintaining gut health (Song et al., 2014; Abdelli et al., 2021; Wang C. et al., 2021). However, very limited data is available regarding HS exposure’s effects on jejunal immune response in yellow-feathered broilers. In the present research, the ELISA and mRNA results indicated that HS exposure elevated the production of pro-inflammatory cytokines (TNF-α, IL-1β, and IL-6) in jejunal mucosa. In accordance with the studies of fast-growing broilers, Song et al. (2017) found that the HS has increased the mRNA expression levels of pro-inflammatory cytokines such as IL-1β and IL-6 in the jejunum of Arbor Acres broilers. Siddiqui et al. (2020) demonstrated that Ross broilers exposed to HS had high relative mRNA expression levels of jejunal pro-inflammatory cytokine genes (*IL-6* and *TNF-α*). At the same time, Al-Zghoul and Saleh (2020) observed that HS exposure up-regulated the jejunal mRNA expression of *TNF-α*, *IL-1β,* and *IL-6*. Furthermore, this study found that HS promoted the jejunal mRNA expression levels of *NF-κB*. The *NF-κB* is widely reported to activate gut inflammatory responses (Sahin, 2015; Liu et al., 2016). Therefore, the findings of this study further confirmed the theory that HS exposure triggers an intestinal inflammatory response in both fast- and slow-growing broilers, which is probably involved in the activation of *NF-κB* signaling.

Gut microbiota homeostasis is critical for eliminating pathogens, maintaining intestinal epithelial integrity, and regulating mucosal immunity in the GIT (Cao et al., 2021). Existing studies have primarily focused on the effect of HS on the hindgut microbiota of broilers and analyzing the role of microbiota in mediating small intestinal health (Patra and Kar, 2021; Yadav et al., 2021). However, the microbiota colonized in the jejunum can directly crosstalk with the barrier integrity and immune function (Gong et al., 2020). Thus, this study detected the jejunal microbiota structure and community using 16S rRNA sequencing. The alpha diversity of jejunal microbiota was not affected by HS exposure. Similar findings from hindgut microbiota were obtained previously. For instance, Liu et al. (2021a) reported that HS exposure had no significant impact on the alpha diversity index of cecal microbiota in yellow-feathered broilers. Wasti et al. (2021b) suggested that there were no obvious changes in cecal microbial alpha diversity such as Shannon entropy and Simpson’s index of Cobb broilers under HS. Moreover, in this study, the jejunal microbial species number was decreased by HS, suggesting that although HS exposure did not alter the alpha diversity, it reduced the species richness of jejunal microbiota in yellow-feathered broilers. Regarding the microbial composition, HS had no significant effects on the relative abundance of microbiota at the phylum, class, order, and family level. Partly consistent with our results, Shi et al. (2019) found that 28 days of HS did not change the cecal microbial abundance at the phylum level. According to Wang G. et al. (2021), there were no significantly different abundances of cecal bacteria at the phylum level in Arbor Acres broilers under HS. On the contrary, in the study of Liu et al. (2021a), there were cecal bacteria with significantly different abundances at the phylum, class, order, and family level in yellow-feathered broilers exposed to HS. Many factors could contribute to the regulation of gut microbiota composition, such as diet, host genetic background, stress intensity, and intestinal segments (Xia et al., 2022), which might explain the inconsistencies. Notably, HS exposure significantly reduced the relative abundance of *Ruminococcus*, *Bdellovibrio,* and *Serratia* at the genus level. *Ruminococcus* can break down the cellulose and ferment glucose, xylose, and polysaccharides, producing beneficial metabolites such as acetate, propionate, and butyrate, which have anti-inflammatory functions in the gut (La Reau and Suen, 2018; Jha and Mishra, 2021). It has been found that the abundance of *Ruminococcus* was reduced in the intestinal inflammatory process of ulcerative colitis and revealed its probiotic and gut health-promoting property (Li et al., 2020). *Bdellovibrio* has bacteriophage-like action to control harmful bacteria (Sockett and Lambert, 2004) and plays a probiotic role in improving gut health (Dwidar et al., 2012). A previous study reported that *Bdellovibrio* enters the intestine and releases peptidoglycan and immune substances through glycanase and peptidase, thereby exerting immunomodulatory activity (Chen et al., 2018). Additionally, *Serratia* is a type of intestinal commensal microbial in animals; the prodigiosin, a metabolite from *Serratia,* which exhibits anticancer and antibacterial functions (Soenens and Imperial, 2020). However, some strains of *Serratia* are pathogenic (Abreo and Altier, 2019); the studies on *Serratia,* including the beneficial and pathogenic strains in poultry intestines, are limited. It is necessary to further isolate and identify the *Serratia* species in the GIT of broilers and clarify their roles in HS response. Based on the potential immunomodulatory and antibacterial effects of the microbiota with significantly different abundances, it could be speculated that HS-induced jejunal immune dysfunction might be related to the reduced levels of *Ruminococcus*, *Bdellovibrio,* and *Serratia*, but the specific interaction analysis is required.

Emerging evidence linking HS to intestinal damage suggests that the gut microbiota might be an under-appreciated mediator of the inflammatory response (Wen et al., 2021). To delineate the interaction of jejunal microbiota and immunity in yellow-feathered broilers, Spearman correlation analysis was performed in this study. At the phylum level, four species of bacteria were significantly associated with immune genes expression, which were positively correlated with anti-inflammatory cytokines and negatively correlated with pro-inflammatory cytokines. *Bacteroidetes* is a common parasitic microbial in the gut; it also showed a negative correlation with intestinal pro-inflammatory cytokines in pigs (Xia et al., 2022). There are few studies on the relationship between *Cyanobacteria*, *Thermi*, *TM7,* and immune response; further research is needed to elucidate this correlation (Whitton and Potts, 2012; Rinttilä and Apajalahti, 2013; Chen et al., 2021). At the genus level, ten species of bacteria were significantly related to the expression of immune genes. Specifically, the abundances of *Ruminococcus* and *Bdellovibrio* were negatively associated with the pro-inflammatory cytokine expression and *NF-κB* signaling; this may be due to the anti-inflammatory and immunomodulatory activities of the metabolites produced by *Ruminococcus* and *Bdellovibrio* (Chen et al., 2018; Li et al., 2020). Besides, *Actinomyces* has been reported to regulate the immune system, especially cellular immunity, and positively correlated with the production of INF-γ (Hötte et al., 2019); this is consistent with our results. The correlation result of *Ruminococcaceae* was similar to the *Ruminococcus.* A previous study also suggested that the *Ruminococcaceae* was involved in regulating inflammatory bowel disease through secondary bile acids (Guo et al., 2020). *Clostridiales* exhibited a significant negative correlation with pro-inflammatory cytokines and *NF-κB* signaling. The *Clostridium butyricum* of *Clostridiales* is a probiotic and has anti-inflammatory properties in broilers (Zhang et al., 2014). Conversely, the *Clostridiales* also contain some pathogenic bacteria species (Paredes-Sabja et al., 2011). Therefore, the role of *Clostridiales* in modulating gut immunity is subject to further verification. Furthermore, the *Bacteroides* showed a similar correlation trend with *Bacteroidetes* at the phylum level. However, other microbiota related to immune genes expression, such as *Caulobacteraceae, Thermus, Sediminibacterium,* and *Sphingomonadales*, their function with immunity is still unclear and needs to be elucidated. Together, integrating the bacterial abundances and correlation analysis, it can be noted that *Ruminococcus* and *Bdellovibrio* may be the key microbially mediated elements of the jejunal inflammatory response during HS. And, dietary supplementation of *Ruminococcus* and *Bdellovibrio* as potential probiotics may alleviate HS-induced intestinal inflammation in yellow-feathered broilers; further validation of this hypothesis would be valuable.

## Conclusion

Four weeks of heat stress at 32.5°C adversely affected jejunal immunity via stimulating the production of pro-inflammatory cytokines such as TNF-α, IL-1β, and IL-6 and activating the *NF-κB* signaling. Heat stress had no significant impact on alpha diversity. The relative abundance of *Ruminococcus*, *Bdellovibrio,* and *Serratia* in the jejunum was decreased by heat stress. Furthermore, *Ruminococcus* and *Bdellovibrio* showed a significant negative correlation with pro-inflammatory cytokines expression. The findings not only provide novel insights into the interaction of jejunal immune response and microbiota, but also contribute to the development of potential nutritional mitigation strategies (probiotics intervention) for the regulation of intestinal immunity in yellow-feathered broilers under heat stress situations.

## Data Availability

The datasets presented in this study can be found in online repositories. The names of the repository/repositories and accession number(s) can be found below: https://www.ncbi.nlm.nih.gov/, PRJNA816650.
